# Quality health services and health research tailored for sexual and gender minority forced migrants: perspectives of migrants with lived experience and health service providers

**DOI:** 10.1186/s12913-026-14676-y

**Published:** 2026-05-22

**Authors:** Maria Gottvall, Rummage Isaac, Stella Kyaligamba Nankinga, Hanna Placid Solimena, Tommy Carlsson

**Affiliations:** 1 The Department of Health Sciences, The Swedish Red Cross University, Huddinge, Sweden; 2 CIRCLE – Complex Intervention Research in Health and Care, The Department of Women’s and Children’s Health, Uppsala University, Uppsala, SE-751 85 Sweden

**Keywords:** Forced migration, Health services research, Sexual and gender minorities, Prioritization

## Abstract

**Background:**

In many settings, sexual and gender minorities experience severe persecution and are left with the only option to flee to another country. The aim of this study was to explore perspectives on what constitutes post-migration quality health services and health research tailored for sexual and gender minority forced migrants, according to the views of migrants with lived experience and health service providers.

**Methods:**

This was a qualitative exploratory study with embedded public contribution through close collaboration with migrants representing the target population. Study participants were recruited through purposeful and snowball sampling (forced migrants *n* = 17, service providers *n* = 20). Semi-structured interviews were transcribed verbatim and analyzed with systematic text condensation.

**Results:**

Open doors and open minds in health services were identified as overarching cornerstones for quality health services. Participants consistently emphasized the need for accessible and welcoming health services, highlighting specific concerns about language barriers and fear of discrimination. A key element was the significance of sufficient awareness and reflection among health professionals who foster client comfort through affirming and individualized care. The importance of upholding confidentiality and privacy was underscored, considering the internalized shame and traumatic experiences represented within the population. High degrees of representation and in-depth considerations of intersectional nuances were identified as overarching cornerstones for health research. A high degree of embedded representation and inclusive approaches in research was highlighted. A need for research that will uncover intersectional mental health burdens and living conditions was articulated, alongside a need for research focusing on interventions that will promote equitable and tailored health services.

**Conclusions:**

This study highlights the urgent need for health services to adapt to the complex and intersecting needs of underserved populations such as sexual and gender minority forced migrants. For quality health services to be achieved, it needs to be grounded in equitable access, intersectional awareness, affirming relationships, and safeguards for safety and confidentiality. Future research needs to prioritize inclusionary and participatory approaches, investigate specific subgroup needs, address multidimensional burdens, and focus on effective and pragmatic support interventions.

**Supplementary Information:**

The online version contains supplementary material available at 10.1186/s12913-026-14676-y.

## Background

Across the world, sexual and gender minorities - including lesbian, gay, bisexual, transgender, and queer persons (often referred to as LGBTQ+) – face societal consequences, pervasive violence, and dangerous threats [1]. Persons whose sexual orientation, romantic relationships, gender identity, and gender expression extend beyond societal norms face proximal stressors related to stigma and prejudice, such as targeted harassment and bullying [2]. Persecution against sexual and gender minorities can take many forms and may culminate in considerable dangers because of the stigmatization and violence enacted by the general population, extremist groups, and family members [3, 4]. While some countries continue to establish laws that protect the rights and safety for sexual and gender minorities, other countries have implemented oppressive legislation, including imprisonment and death penalties, against them. Combined, societal and state-sponsored oppression and marginalization have a substantial impact on the mental health and wellbeing of sexual and gender minority individuals [5]. When compared to heterosexual and cisgender counterparts, sexual and gender minorities are at higher risk of experiencing mental health burdens, including depression, anxiety, self-harm, and suicide ideation [6–9]. The lifelong exposure to oppression can involve various forms of sexual violence, torture, psychological abuse, and witnessing the murder of loved ones as an enactment rooted in heterosexism, cisgenderism, and anti-trans sentiment [3, 4]. The lack of safety and exposure to threatening situations can become so severe that the only viable option is to flee and seek protection in another country [4]. Indeed, many countries acknowledge fear of persecution based on sexual orientation and gender identity as a valid reason to be granted asylum [10].

With an all-time high number of persons who are forced to flee because of persecution and human rights violations, forced displacement is an urgent global public health crisis [11]. Forced migration is a significant life event involving a risk of development of various mental health disorders, including posttraumatic stress, depression, and anxiety [12–14]. It also involves a risk of physical non-communicable chronic conditions, such as cardiovascular diseases [15] and diabetes mellitus [16]. Intersectionality concerns the overlapping systems of hegemonies related to power and oppression that shape privileges and disadvantages in society. Through an intersectional lens, being both a forced migrant and self-identifying as a person of sexual and gender minority identity can result in mental health burdens due to compounded minority stress and oppression [2]. For example, heterosexism, genderism, and anti-trans sentiment can intersect with racism and ethnocentrism to shape societal disadvantages that impact the health and wellbeing of sexual and gender minority forced migrants. Research indicates that sexual and gender minority forced migrants experience a range of complex mental health burdens following their arrival [17, 18]. Carrying the burden of trauma, sexual and gender minority forced migrants experience various symptoms of post-traumatic stress including intrusive thoughts, nightmares, avoidance, and hyperarousal [3, 18, 19]. Worries and fears related to the asylum claim and their safety while residing in the host country, alongside the safety of people left in their country of origin, highly impacts their mental health [3, 17]. Further, sexual and gender minority forced migrants experience significant mood disturbances and existential loneliness [3].

While host countries have a responsibility to support forced migrants, barriers to access health services negatively impact their health and wellbeing [20, 21]. Living undocumented is associated with further barriers, such as high costs, legal restrictions, and ambiguous policy messages [22]. In addition to the barriers to access health services encountered within the wider migrant population, sexual and gender minority forced migrants experience specific barriers related to fears, internalized stigma, and shame [23, 24]. When able to access health services, forced migrants, as well as persons of sexual and gender minorities, are at risk of encountering discrimination, emphasizing the importance of developing culturally sensitive support [25, 26]. Experiences of heterosexism, cisgenderism, and anti-trans sentiment in healthcare settings further call attention to the need for the continued development of high-quality care based on health equity and nondiscriminatory practices [23]. However, little is known how to prioritize and structure health services to further accommodate the needs among sexual and gender minority forced migrants. Research addressing the health of sexual and gender minority individuals lack sufficient consideration of people finding themselves at the intersections of multiple forms of marginalization, including a lack of research about the needs of migrants within this population [27]. The overall lack of consideration of sexual orientation and gender identity in research further drives the underrepresentation [28].

Against this backdrop, more research is needed to increase the understanding of how to develop and deliver quality health services and health research for sexual and gender minority forced migrants. To address this gap in knowledge, we set out to conduct a qualitative study encompassing the views of sexual and gender minority forced migrants and health professionals who support them. Specifically, the aim of this study was to explore perspectives on what constitutes post-migration quality health services and health research for sexual and gender minority forced migrants.

## Methods

### Study design

This was a qualitative exploratory study with embedded public contribution. Two individuals with lived experience as sexual and gender minority forced migrants, as well as one clinical health professional with experience of supporting forced migrants (specialist nurse), contributed as equal research partners. The purpose behind the public contribution was to reach democratized findings based on lived experiences and diverse perspectives [29].

Experts by lived experience as sexual and gender minority forced migrants were involved throughout the complete research cycle as equal partners in the research team, engaged from conceptualization and planning to analysis and dissemination. The experts were employed at the university and did not act in the capacity of study participants. They had regular working hours at the host university for the research team and participated in daily meetings together with the researchers. Through close collaboration with the senior researchers and the clinical health professional, the experts: (i) planned study procedures, including recruitment strategies, (ii) drafted and refined the interview guide, (iii) engaged in the data collection when appropriate, (iv) transcribed audio recordings of interviews, (v) co-analyzed the interviews throughout all steps of the analysis, (vi) wrote drafts of the qualitative findings, and (vii) reviewed and provided feedback on the draft of the manuscript.

### Participants and recruitment

The sample included persons with lived experience as sexual and gender minority forced migrants, as well as health professionals with experience of supporting forced migrants and sexual and gender minority individuals. Between October 2024 and February 2025, participants were recruited through purposeful and snowball sampling utilizing the established professional and community networks of the research team. We strived to achieve maximum variation within the sample regarding background characteristics such as gender identity, sexual orientation, and health profession. Throughout the recruitment process, we scrutinized the background characteristics of the included participants to identify gaps in diversity which needed to be addressed by targeted recruitment via our networks. For example, we employed purposeful sampling to reach transgender and non-binary persons, as well as reaching migrants with varied countries of origin. The public contributors were closely involved in the recruitment to strive for diversity, acting as community liaisons towards forced migrants.

Due to the exploratory aim of this study, an open approach was applied regarding inclusion criteria. For forced migrants, they needed to view themselves as a forced migrant, self-identify as a sexual and/or gender minority individual, and be 18 years of age or older. For health professionals, they needed to have any profession involved in providing support within healthcare settings. While no criteria were applied for language proficiency, none of the forced migrants required an interpreter for the interview. The final sample consisted of 37 participants (forced migrants *n* = 17, health professionals *n* = 20). Table 1 presents the sample characteristics. All participants were given written and oral information about the study, and all signed a written informed consent document before the data collection. Participants received a gift card of SEK 250 following the interview.

**Table 1:** Sample characteristics of forced migrants (*n* = 17) and health professionals (*n* = 20)

Characteristic		Forced migrants	Health professionals
Age	20–29	1	-
	30–39	8	4
	40–49	8	9
	50–59	-	4
	> 59	-	3
Gender identity	Cisgender man	8	6
	Cisgender woman	4	11
	Transgender man	1	-
	Transgender woman	2	-
	Non-binary/ genderfluid/genderqueer	2	3
Region of origin	Africa	8	-
	Asia	7	-
	Europe	2	20
Sexual orientation	Gay	8	-
	Lesbian	4	-
	Bisexual	2	-
	Straight/heterosexual	2	-
	Queer	1	-
Profession	Midwife	-	5
	Social worker/Counsellor	-	5
	Psychologist	-	4
	Nurse	-	2
	Physician	-	2
	Physiotherapist	-	1
	Occupational therapist	-	1

### Data collection

Individual semi-structured interviews were conducted face-to-face (*n* = 10), via telephone (*n* = 1) or through the digital video conferencing tool Zoom (*n* = 26), depending on the preferences and availability of the participant. A semi-structured interview guide (Table 2 and Supplemental File 1) was utilized to ensure that the interviews covered the aim of the study while simultaneously allowing for flexibility and probing questions as needed. The guide was developed for this study and in close collaboration between researchers, experts by lived experience as sexual and gender minority forced migrants, and clinical health professionals. Questions included what the participants felt is included in appropriate care for the target population, and further, which areas they perceived that research should prioritize regarding the health and wellbeing of the target population. Clarifying and probing questions were used throughout the interviews to explore the perspectives of the participants. For example, questions such as ‘*Can you please explain more*?’ and ‘*Is there anything else you want to add?*’ were asked to explore the aim in greater depth.

**Table 2:** Semi-structured interview guide

Topic	Main question	Follow-up questions
Components of adequate and appropriate care	What is appropriate care for sexual and gender minority forced migrants?	How should forced migrants be supported in care?
		What knowledge do health professionals need regarding this population?
Research that should be prioritized to address health and wellbeing	What do you think future research should investigate regarding sexual and gender minority forced migrants, when it comes to health and wellbeing?	What health outcomes are relevant to assess in future research regarding these persons?
		What support interventions such as aids, tools, or treatments are relevant to develop and evaluate in future research addressing the health and wellbeing of these persons?

The length of the interviews ranged between 24 and 79 min (Mean: 38 min). Interviews were attended by either two research team members, in which one acted as an observer and the other as interviewer (*n* = 22 interviews), or by a single senior researcher experienced in qualitative interviews (*n* = 15 interviews). All team members involved in the interview introduced themselves before any data collection was initiated, and participants had the option to decline the presence of the observer. To promote comfort during interviews, participants had the opportunity to choose the location for the interview and could pause the recording when so desired. All interviews were audio-recorded and transcribed verbatim.

### Data analysis

Transcribed interviews were analyzed in a collaborative process according to the steps in systematic text condensation, which is an exploratory and descriptive method for cross-case thematic analysis of qualitative material [30]. The method is a pragmatic approach to exploring qualitative interviews in a stepwise manner, fitting for both novice and more experienced analysts, making it a suitable alternative for co-analysis together with experts by lived experience. All transcripts were read carefully to identify preliminary themes. Code groups were derived from these preliminary themes and meaning units were placed in the code groups accordingly. Analysis was further refined as meaning units were placed in subgroups and condensates were produced, acting as artificial quotes covering the content of each subgroup. Synthesized statements were written based on the condensates, and illustrative quotes were derived from the raw data. Lastly, category headings were generated, providing statements of the code groups.

The analysis was an iterative process involving a team of researchers, one clinical representative, and two experts by lived experience as sexual and gender minority forced migrants. All team members were engaged in all steps of the analysis and collaborated throughout the process to produce exhaustive findings grounded in the data. Experts by lived experience were actively involved in all analytic steps, spanning from the identification of preliminary themes up until finalization of category headings and writing of the results. Please note that in interviews, the term “LGBTQ+” was often used (“HBTQ+” in Swedish), which is a common abbreviation for lesbian, gay, bisexual, transgender and other queer identities or expressions.

Throughout the analytic process, the research team strived to interpret the data from diverse perspectives, drawing from their positions and backgrounds as experts by lived experience, health professionals, and researchers. The team worked collaboratively in all steps of the analysis and continuously scrutinized their findings in an iterative manner by going back and forth between the raw data and the analysis. Reflexive discussions were held regularly during the analysis to contest the findings and search for diverse perspectives. The researchers are two senior lecturers (PhD’s and Associate professors) with experience of qualitative methods. The first author is a Swedish-born cisgender woman who is a nurse and a midwife, whereas the last author is a Swedish-born genderqueer person who is a specialist nurse and a midwife. Both contributed to the analysis by providing scientific guidance, with a theoretical and research-based perspective on the data. The clinical representative is a Finish-born cisgender woman who is a specialist nurse in psychiatric care, with clinical experience of supporting forced migrant women with traumatic experiences. She contributed to the analysis by providing a clinical perspective on the data, with a focus on mental health and support in the Swedish context. The two experts by lived experience, who are forced migrants representing the target population, contributed to the analysis by providing valuable insights and expertise into the lived experience of the target population. Drawing from their insights into the community from an insider perspective, their involvement shaped the interpretation by grounding the findings in a lived experience understanding.

## Results

For quality health services to be achieved, findings showed that it needs to involve open doors and open minds. For quality health research to be prioritized successfully, participants articulated a need to involve a high degree of representation and nuances taking intersectional aspects into account. The findings are elaborated in the following sections. Table 3 presents an overview of the qualitative findings.

**Table 3:** Overview of the qualitative findings

Category heading	Code group
Open doors and open minds in clinical settings: cornerstones for quality health services	Unlocking the barriers for easy and equitable access to health services
	Acknowledging the entire person through awareness and reflections among health professionals
	Fostering client comfort through affirming and individualized care
	Ensuring safe spaces in care by upholding confidentiality and privacy
The need for representation and intersectional nuances: health research priorities	Embedding multi-level representation and inclusive approaches in research
	Uncovering intersectional mental health burdens and living conditions through research
	Researching interventions for equitable and tailored health services

### Open doors and open minds in clinical settings: cornerstones for quality health services

Participants emphasized a variety of cornerstones needed to achieve quality health services for sexual and gender minority forced migrants. An overarching necessity was equitable and facilitated access to health services, reducing the barriers that forced migrants and persons of sexual and gender minorities encounter. When migrants are indeed able to access health services, intersectional awareness and reflexivity among health professionals providing affirming support was considered essential. The importance of establishing safety and confidentiality during clinical encounters was also identified as a cornerstone for quality health services.

### Unlocking the barriers for easy and equitable access to health services

Accessible health services emerged as a foundation for addressing the challenges faced when trying to navigate an unfamiliar and complex health system. To improve access, participants felt that society needs to reduce the language barriers encountered by forced migrants. They also called attention to the potential positive effects of easy appointment booking through digital systems and the option for choosing anonymous drop-in visits. The importance of readily available and free health services was emphasized, including services catering to sexual health and the needs of the target population.

*I think that good care for LGBTQ+ migrants partly is accessible in the way that it’s easy to get a hold off*,* easy to reach*,* easy to find online*,* and that there is information at least in English and maybe even in other languages.* (Health professional 1)

Mitigating the barriers unaccompanied minors and undocumented migrants encounter when trying to access health services was considered essential. This included addressing the fear of being reported to the migration authorities that undocumented migrants can experience, hindering them access to needed healthcare. To enhance access to health services, participants expressed a need for improved information dissemination on how to access health services. Further, they suggested improved coordination between health services and non-governmental organizations, to increase the uptake of health services among migrants.

[It should be] *as easy as possible to access health centers*,* even when we are not officially registered* […] *I know that there are people who have not been lucky with that*,* and it has been hard to access the health services. Because they are really afraid what is going to happen when* [they] *go and present* [themselves] *to the health centers.* (Migrant 1)

The findings in this code group call attention to the need for further improvement in structures that can make health services more accessible and easier to find for forced migrants. Participants highlighted a range of barriers that forced migrants encounter when trying to access health services, suggesting interventions that may enhance the service uptake among migrants.

### Acknowledging the entire person through awareness and reflections among health professionals

Sufficient intersectional awareness and reflexivity among health professionals were considered essential. This encompassed a holistic understanding about the spectrum of diverse sexual orientations, gender identities, and gender expressions. It also included awareness about stereotypes, norms, and assumptions related to identities, relationships, and family structures. According to the participants, health professionals should all have sufficient awareness about health burdens and needs within the target population, ranging from pre-migration trauma exposure to post-migration health risks. Sufficient awareness about the rights to access health services as a forced migrant was also emphasized.

[Health professionals] *don’t have any knowledge in LGBTQ*,* transgender*,* with all those they know nothing. I’ll say people are different*,* people have different needs. As a transgender person*,* my need is different from a cis-gender man or a trans man.* […] *They need to have basic knowledge about us and our needs.* (Migrant 2)

*One other thing I would say is something to do with trauma-informed care. I think you know that because… Health care personnel should know that we people that come here could have been tortured*,* discrimination from our countries. So*,* if you are going to receive me you must be competent enough to know that this person has gone through a lot.* (Migrant 3)

Self-reflection among health professionals, particularly in relation to their own identities and preconceptions, was seen as key to reducing bias during clinical encounters. Suggested strategies to promote awareness among health professionals included structural processes that promote lifelong development, education on diverse perspectives outside normative frameworks, and certification in supporting sexual and gender minorities. Participants stressed the importance of an open work environment where service providers can discuss mistakes, ask for advice, and have sufficient clinical time for each client. The need for long-term quality projects and cultural diversity among health professionals was also articulated.

*I think we need to create a more open and conversational work environment* [in health services] *where there is opportunity to discuss your mistakes or ask for advice from others.* […] *I think that there are a lot of taboos to talk about it. So*,* that we in some way*,* it is my wish*,* that we can promote a work environment where it’s possible to learn from each other.* (Health professional 2)

The findings in this code group call attention to the importance of continued awareness and reflexivity among health professionals, so that they can approach each client encounter in an individualized and open manner. Participants emphasized that health professionals need sufficient awareness about a range of aspects related to both forced migration as well as identities and expressions that extend beyond normative frameworks in society.

### Fostering client comfort through affirming and individualized care

Affirming support was considered an essential part in quality health services. Based on the perspectives of the participants, health professionals must promote feelings of comfortability without instilling fear of judgment among those seeking professional support. Thus, they stressed the importance of an inviting and warm health system, including health professionals who show friendliness, validation, respect, and inclusiveness. This was considered especially important given the shame experienced among people within the target population.

*I think people that are going to be given care should be recognized in terms of validation*,* that means the people there must be ensuring respect and value in their interaction.* (Migrant 3)

Another central aspect according to participants was building trust and forming an alliance with patients and clients. This included the importance of recognizing individual needs, challenges, and preferences by avoiding decisions based on stereotypes and assumptions. Empathy, active listening, and a person-centered approach were seen as promoters of open communication. Migrants desired health professionals who asked open-ended questions and respected their identities, while simultaneously taking cultural dimensions of sexuality and sex into account.

*I think a space of inclusivity is very important* […] *if you see me*,* you will say this is a beautiful handsome man*,* which I am not. That is not how I identify myself. I think the healthcare professionals have to have the reflection of how to ask somebody ‘how can I identify you’.* (Migrant 2)

The findings in this code group call attention to the importance of client comfort when supporting sexual and gender minority forced migrants. Participants highlighted that health professionals need to create an inviting and non-judgmental clinical atmosphere where migrants feel comfortable and seen.

### Ensuring safe spaces in care by upholding confidentiality and privacy

Safety and confidentiality were considered key in building trust and promoting disclosure in clinical settings. This required health professionals to approach sensitive issues appropriately and provide clear assurance of confidentiality. It also included the importance of ensuring patient privacy and comfortability by informing them that they will not be reported to authorities when seeking healthcare.

*I think the biggest support would be confidentiality*,* because you will find most of these migrants fear to even go for health services because someone has this fear. Remember*,* they have those traumas from back home.* (Migrant 4)

Providing clear information about the rights to health care and utilization of interpreters when needed were considered paramount. Participants emphasized continuity of care and suggested a need for a designated contact person who coordinates care, provides guidance, and offers support. Acknowledgement of the power imbalance that exists between health professionals and those seeking care was seen as vital for reaching equitable clinical encounters.

*Within healthcare*,* there will always be a hierarchy in encounters. It’s sort of the basis that one ends up in a position of dependency as a patient.* […] *We have a very big obligation to do everything we can to make the person feel as safe as possible.* (Health professional 3)

The findings in this code group call attention to the importance of upholding confidentiality and safety when supporting this population, as clients may experience fears and shame related to their identity as well as their migration status. Participants highlighted that health professionals need to provide clear information and strive for continuity of care, to enhance trust and reduce power imbalances in clinical encounters.

### The need for representation and intersectional nuances: health research priorities

Participants expressed the need for future health research to focus on three overarching areas. Firstly, participants called attention to the importance of ensuring adequate representation and inclusive approaches in research methods moving forward. Secondly, they wanted more research that addresses the living conditions, health burdens, and structural inequalities experienced within the target population. Thirdly, exploration of interventions aiming to enhance equitable and tailored health services was suggested as an area for prioritization.

### Embedding multi-level representation and inclusive approaches in research

This code group was only generated from data based on health professionals, who called attention to the importance of ensuring a diverse representation in health research. They underlined that the specific needs of different subgroups can vary widely, making it essential for research to ask questions about sexual orientation and gender identities to allow in-depth exploration and intersectional insights. It was noted that White and highly educated people are disproportionally overrepresented in health research, necessitating research that ensures more diverse samples and exploration of the health of underserved subgroups. For example, the needs of transgender persons were articulated as an understudied area in need of further research.

[Interviewer: How can research progress forward when it comes to the health and wellbeing of this target population? ] *I think it is important to separate LGBTQ*,* that there is a difference in needs. That we find women with migration experience*,* they are seldomly heard.* […] *When we look into healthcare and healthcare interventions*,* you may need to seek care because of your trans identity.* […] *Trans people should get their own research.* (Health professional 5)

To ensure inclusive approaches in research, participants stated the need for research focusing on reaching forced migrants who may otherwise be overlooked. Finding ways to connect migrants with needed healthcare was considered a vital step in future research. In addition, public contribution was pointed out as a strategy to foster trust in research among migrants. To develop effective research interventions, participants suggested tailored recruitment strategies and placing the needs of the target population at the center when identifying research questions.

*In terms of which interventions are needed. I think that we need to let the target population define it for themselves. How should I know what they need. I can base it on theory and on what I know*,* but it’s not based on them and their backgrounds. The existing theories are really centered around Western countries. Personally*,* I am for participatory action research.* (Health professional 2)

The findings in this code group call attention to the importance of achieving diversity and representation in health research throughout the research cycle. Participants emphasized the need to further prioritize tailored recruitment strategies and to strengthen public contribution in research.

### Uncovering intersectional mental health burdens and living conditions through research

Intersectional mental health burdens, including the health impact of disadvantaged living conditions and multi-level vulnerabilities in a life course perspective, were considered prioritized areas. This included migrants’ experiences of structural inequalities, discrimination, pre-migration trauma, acclimatization to a new country, interactions with authorities, and financial challenges. Research focusing on post-migration mental health was especially highlighted, including minority stress, identity concealment, shame, suicidality, and potential subgroup differences. Specifically, the risk of exposure to violence and hate crimes was articulated. The participants stated a need for research investigating the impact of loneliness and what contributes to resilience, including social support interventions.

*I meet a lot of migrants who are LGBTQ+*,* many of them have been exposed to much violence previously in their lives*,* in other countries as well as in Sweden. Research [is needed] about the vulnerability to violence - both hate crimes and violence in general - but also domestic violence*,* honor-based violence*,* violence in an honor context.* (Health professional 6)

Research about the health burdens related to migration stress, seeking asylum, and facing potential deportation was articulated among the participants. They suggested a need to develop methods that will ensure case officer competence and promotion of feeling safe during the asylum process. A need for the development of strategies to promote migrants’ establishment in the host country was also articulated, including the promotion of employment attainment, healthy lives, safe accommodation, and language skills. Research focusing on tailored support for undocumented migrants was specifically highlighted, to promote peaceful lives free from substance use, sex work, abuse, and violence.

*I’ve had so many difficulties when I came to Sweden. My accommodation problems*,* inclusion problems*,* because of language barriers.* […] *When migrants come here*,* we should conduct research of what they want and how they are living.* (Migrant 6)

The findings in this code group call attention to the importance of research that prioritizes a multi-level framework taking into consideration the vulnerable situations that sexual and gender minority forced migrants encounter in the post-migration phase. Participants suggested the prioritization of uncovering structural disadvantages and mental health burdens experienced within this population.

### Researching interventions for equitable and tailored health services

Participants expressed a need for research that will promote health through tailored interventions providing support for the target population. One area for intervention research addressed by participants included the development of new strategies to provide sexual and gender minority forced migrants with clear information in different languages, including information about the health system, how to access health services, sexual health, and common health burdens. A need for research that explores interventions to enhance the detection of sexually transmitted infections, and research which addresses health service accessibility for underserved populations, was emphasized. Health professionals articulated the need for research on how to improve collaboration between health services and different interest holders, to improve accessibility to health services.

[Interviewer: You can also do research that is focused on support interventions.] *I think that healthcare*,* which is also publicly funded*,* should be more outward-oriented. […] To reach people that we don’t reach otherwise.* (Health professional 4)

Another area for prioritization included the establishment of strategies to promote awareness and knowledge among health professionals. The identification of clinical approaches that promote a safe, friendly and non-judgmental clinical setting was emphasized. Moreover, participants expressed a need for research investigating the long-term health impact of specific mental health treatment protocols, such as compassion-focused therapy and acceptance and commitment therapy, as well as a need for studies assessing the health impact of undergoing gender-affirming hormone therapy.

[Health professionals] *do not discriminate*,* but it is very rare that they think more than male and female. So*,* to my experience*,* I am a transgender woman*,* identify as a woman*,* there my needs will be different from a biological woman. So*,* in that case*,* the healthcare professional is confused. So*,* this is what I think the research needs. And the result could lead to better training and development of healthcare professionals.* (Migrant 5)

The findings in this code group call attention to the importance of research that prioritizes health-promoting interventions that will enhance the access and efficacy of healthcare regimes. Participants suggested the prioritization of interventions that will improve information dissemination, collaboration with interest holders, awareness among health professionals, and the evaluation of mental health support interventions.

## Discussion

The aim of this study was to explore perspectives on prioritization of what constitutes post-migration quality health services and research addressing the health and wellbeing of sexual and gender minority forced migrants. Our findings highlight the importance of equitable and inclusive approaches in both health services and within health research.

For health equity to be achieved, all people must have fair and just opportunities to achieve healthy lives. This requires the elimination of disparities adversely impacting the health of excluded and marginalized groups [31]. A breadth of research calls attention to the global need to improve the structural disadvantages impacting the health and wellbeing of forced migrants [32, 33]. Research indicates that more than one in five migrants choose not to seek care despite needing it [34], and that they are reluctant to seek healthcare due to fears of deportation and practical barriers [23, 34, 35]. Our participants called attention to this risk and articulated a need for further interventions to mitigate this. The unmet health needs of migrants call attention to the fact that health services fail to reach those in need of support. Identified strategies which have the potential to reduce barriers are the provision of interpreting services, multilingual instructions, and multilingual medical assistance telephone lines [36]. These interventions need further attention in research. Our findings further emphasize a need for policy changes that will promote equitable access to health services for underserved populations experiencing intersectional disadvantages. Specifically, our participants expressed a need for policies that will reduce multi-level barriers to health services, improve interpersonal communication and support from health professionals, and ensure confidentiality in clinical settings.

Our findings give further strength to previous studies showing that sexual and gender minority forced migrants encounter discrimination and non-affirming behaviors when seeking support from health services [23, 37]. This illustrates the complex systems that exist related to power and privilege that influence interpersonal communication between patients and health professionals. We identified a need for research prioritizing the promotion of awareness and knowledge among health professionals as well as clinical strategies promoting client comfortability. Evidence indicates that training programs for health professionals have the potential to enhance knowledge and affirming practices when supporting migrants [38] as well as sexual and gender minority populations [39]. However, less is known about training programs taking into account intersectional aspects, and further, there is a need for the development and refinement of educational content to address diversity and intersectional perspectives [40]. For example, the participants highlighted the need for enhanced awareness of forced migrants’ rights to access healthcare, the health burdens of forced migrants as well as sexual and gender minority individuals, and an increased understanding of the various identities and expressions that extend beyond societal norms. These findings echo what students and lecturers at nursing education describe, with a lack of mandatory inclusion contributing to low awareness on how to provide culturally sensitive care [41]. Further research is warranted to develop interventions that will promote intersectional awareness and knowledge among health professionals.

According to several reviews, an increasing number of descriptive studies have been conducted to understand the life situation of sexual and gender minority forced migrants. Combined, these reviews call attention to widespread social disadvantages contributing to poor health and wellbeing [4, 17, 23, 24, 42]. Through this study, we wanted to generate findings that will help guide research forward, based on the perspectives of both migrants from the target population as well as health professionals supporting them. Participants highlighted the need for tailored interventions, moving beyond descriptive research. Indeed, only a small proportion of research focusing on sexual and gender minority populations develop and test interventions aiming to improve their mental health, and there is a need for further research investigating how to eradicate health disparities [43]. Such research requires careful consideration, so that interventions to as high extent as possible correspond to the needs of the target population. Wasteful health research due to a mismatch between the interests of the community members and researchers is an acknowledged problem [44]. Our participants suggested public contribution and enhanced representation in research as strategies to place the needs of the target population at the center. Public contribution has the potential to bridge gaps between research and target populations, enhancing the relevance and appropriateness of methods utilized throughout the research cycle [45]. Taken together, our findings give further weight to the need for tailored and co-developed support interventions.

There are methodological limitations to this study that need consideration when interpreting the findings. We utilized purposive and snowball sampling to strive for diversity among our participants. The final sample consisted of 37 participants and was varied regarding age, gender, profession, and sexual orientation. The final number of participants was determined through assessment of thematic saturation. However, none of the participants originated from North America or South America, and all the health professionals originated from Europe. Thus, the transferability of the findings has limitations regarding countries of origin. Research team members experienced in qualitative data collection conducted face-to-face and digital semi-structured interviews with the aid of an interview guide, to stay close to the study aim. While we argue that the interviews provided rich data, we cannot disregard the possibility that some information might have been lost due to communication issues such as when the interview was conducted in a digital setting. Moreover, qualitative analyses are always to some extent influenced by the preconceptions of the analysts [46]. To address this, the researchers in this study engaged in close collaboration with sexual and gender minority forced migrants as well as clinical health professionals during all phases of the analysis, to approach the data from varied perspectives. We believe that our collaborative analytic approach enhanced the confirmability and authenticity of the findings.

## Conclusions

This study highlights the urgent need for health services to adapt to the complex and intersecting needs of underserved and marginalized populations, such as sexual and gender minority forced migrants. For quality health services to be achieved, it needs to be grounded in equitable access, intersectional awareness, affirming clinical relationships, and strong safeguards for safety and confidentiality. Future research needs to prioritize inclusionary and participatory approaches, investigate specific needs of subgroups, address the multidimensional burdens experienced within the target population, and focus on developing effective and pragmatic support interventions. To improve health equity, health services and research must actively prioritize addressing the voices and needs of intersectionally disadvantaged and underserved populations, including this target population.

## Supplementary Information

Below is the link to the electronic supplementary material.

Supplementary Material 1

## Data Availability

The data is not available due to reasons related to ethical considerations and confidentiality.
