# Supplementary material for: Quality health services and health research tailored for sexual and gender minority forced migrants: perspectives of migrants with lived experience and health service providers
Source: BMC Health Serv Res. 2026 May 22;26:739. doi: 10.1186/s12913-026-14676-y (PMC13202932; doi:10.1186/s12913-026-14676-y)
Supplement: Supplementary file 1 — Supplementary Material 1 [file 12913_2026_14676_MOESM1_ESM.pdf]

Supplemental File 1. Semi-structured Interview guide.

Introduction

This project aims to increase knowledge on how healthcare, education, and research can meet the needs of sexual and gender minority forced migrants in Sweden. Sexual and gender minority include LGBTQ+, defined here as lesbian, gay, bisexual, transgender, and people with other queer expressions or identities. The purpose of the study is to explore how support and future research should be designed to meet the needs of these individuals. Do you have any questions? If anything comes up, like you are unsure about some words or some questions, feel free to ask and we can explain it more. Is it okay if we get into the questions?

Interview guide

Topic	Main question	Follow-up questions
Components of adequate and appropriate care	What is appropriate care for sexual and gender minority forced migrants?	How should forced migrants be supported in care?
		What knowledge do health professionals need regarding this population?
Research that should be prioritized to address health and wellbeing	What do you think future research should investigate regarding sexual and gender minority forced migrants, when it comes to health and wellbeing?	What health outcomes are relevant to assess in future research regarding these persons?
		What support interventions such as aids, tools, or treatments are relevant to develop and evaluate in future research addressing the health and wellbeing of these persons?

Examples of probes & further follow-up questions:

Can you give an example of what you just said?
Can you tell me more about that?
Could you please elaborate more?
